# Supplementary material for: Evolution of protein-coupled RNA dynamics during hierarchical assembly of ribosomal complexes
Source: Nat Commun. 2017 Sep 8;8:492. doi: 10.1038/s41467-017-00536-1 (PMC5591316; doi:10.1038/s41467-017-00536-1)
Supplement: Supplementary file 1 — Supplementary Information [file 41467_2017_536_MOESM1_ESM.pdf]

SUPPLEMENTARY INFORMATION

Evolution of protein-coupled RNA dynamics during hierarchical assembly of ribosomal complexes

Sanjaya C. Abeysirigunawardena,[1,a‡] Hajin Kim,[2, 3‡] Jonathan Lai[5], Kaushik Ragunathan,[4,b] Mollie C. Rappé[1], Zaida Luthey-Schulten[5], Taekjip Ha,[1,4,6,7]* Sarah A. Woodson[1]*

[1]T.C. Jenkins Dept. of Biophysics, Johns Hopkins University, 3400 N. Charles St., Baltimore, MD 21218 USA, [2]School of Life Sciences, Ulsan National Institute of Science and Technology, Ulsan 44919, Republic of Korea, [3]Center for Genomic Integrity, Institute for Basic Science, Ulsan 44919, Republic of Korea, [4]Department of Physics, Center for the Physics of Living Cells and Institute for Genomic Biology, University of Illinois at Urbana-Champaign, Urbana, IL 61801, USA, [5]Department of Chemistry, University of Illinois at Urbana-Champaign, 600 S. Mathews Avenue, Urbana, IL 61801, USA, [6]Department of Biophysics and Biophysical Chemistry and Department of Biomedical Engineering, Johns Hopkins University, and [7]Howard Hughes Medical Institute, Baltimore, MD 21205, USA. Baltimore, MD 21205 USA.

Supplementary Figures 1-5 and Figure Legends

SUPPLEMENTARY FIGURE 1

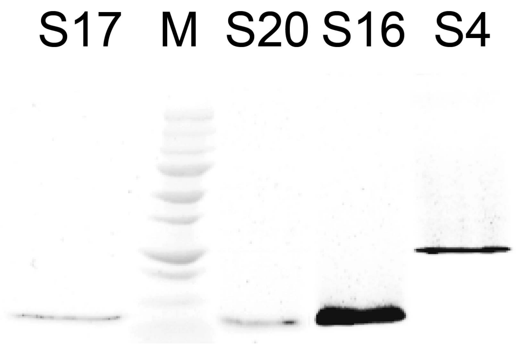

Supplementary Figure 1. Purification of recombinant ribosomal proteins for labeling. Protein variants with single cysteines were prepared as described in Methods and analyzed by SDS-PAGE and Coomassie stain. Proteins were uS17 (S17), markers (M), bS20 (S20), bS16 (S16) and uS4 (S4).

SUPPLEMENTARY FIGURE 2

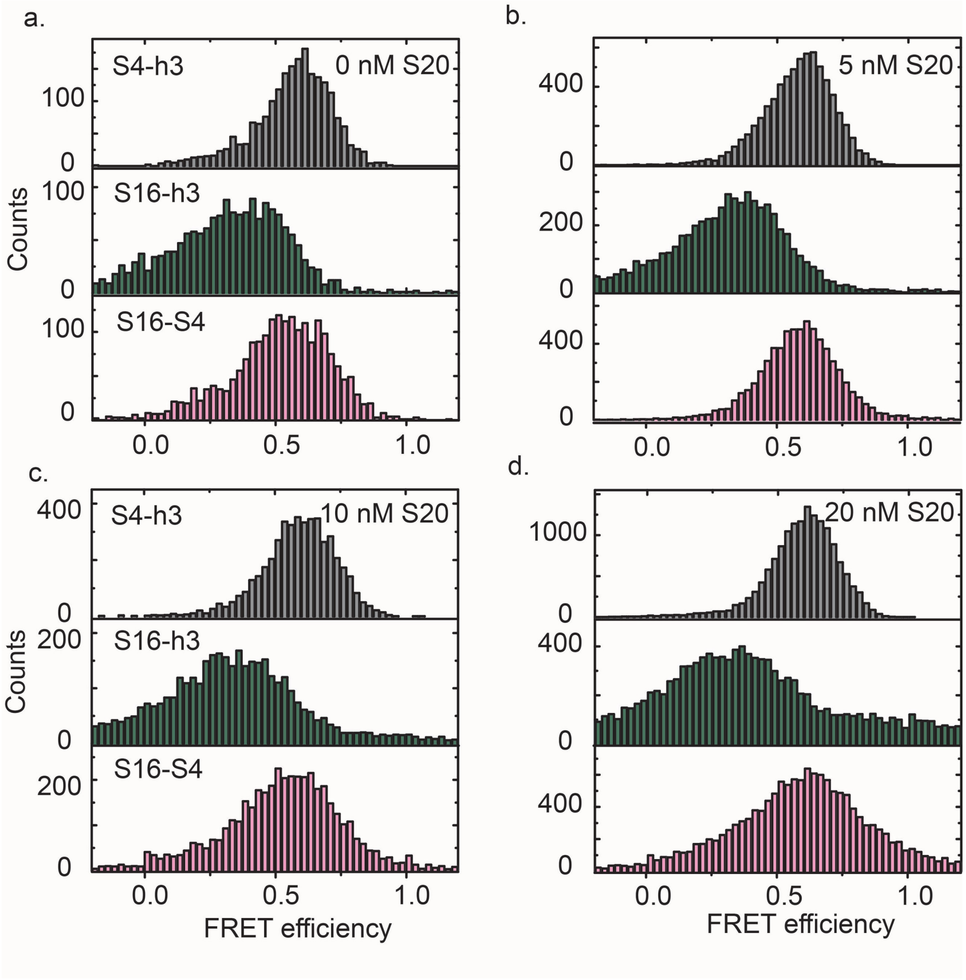

Supplementary Figure 2. Population of FRET intensities in three-color single molecule TIRF experiments. 16S 5′ domain complexes with three labels, S16-Cy3•S5-Cy5•5′domain-Cy7 were immobilized, and the FRET efficiency between each dye pair obtained from the intensities upon alternating Cy3 (green) and Cy5 (red) excitation. FRET population histograms are constructed for Cy5-Cy7 FRET (top panels), Cy3-Cy7 FRET (middle panels) and Cy3-Cy5 FRET (bottom panels) at various concentrations of unlabeled protein S20. All complexes in buffer containing 20 mM $MgCl_2$.

SUPPLEMENTARY FIGURE 3

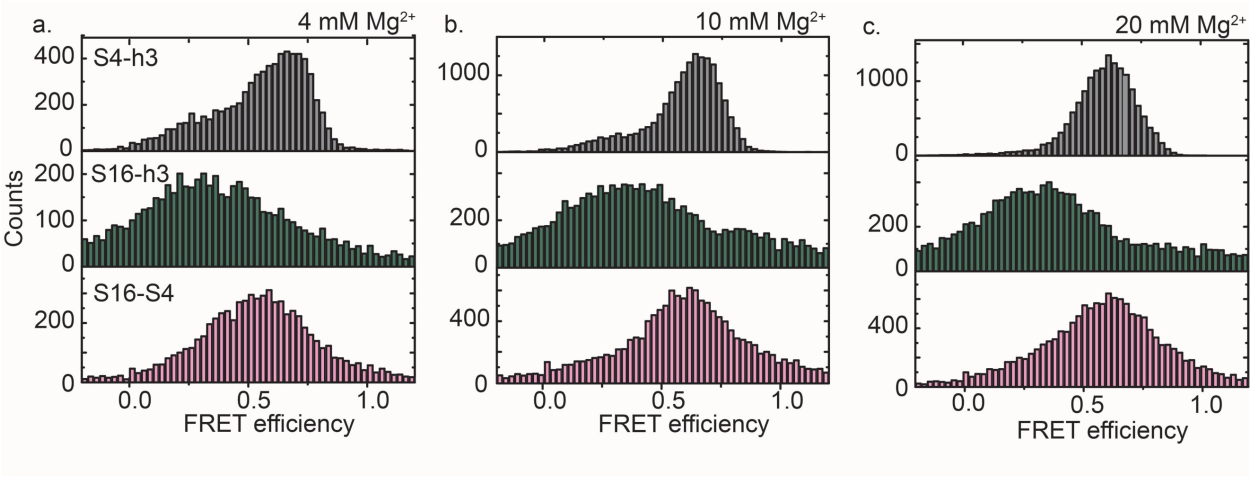

Supplementary Figure 3. Mg^{2+}-dependence of 5′ domain conformation. FRET histograms for Cy5-Cy7 FRET (top panels), Cy3-Cy7 FRET (middle panels) and Cy3-Cy5 FRET (bottom panels) for S4Cy5-RNACy7-S16-Cy3-S20 complexes at various Mg^{2+} concentrations.

SUPPLEMENTARY FIGURE 4

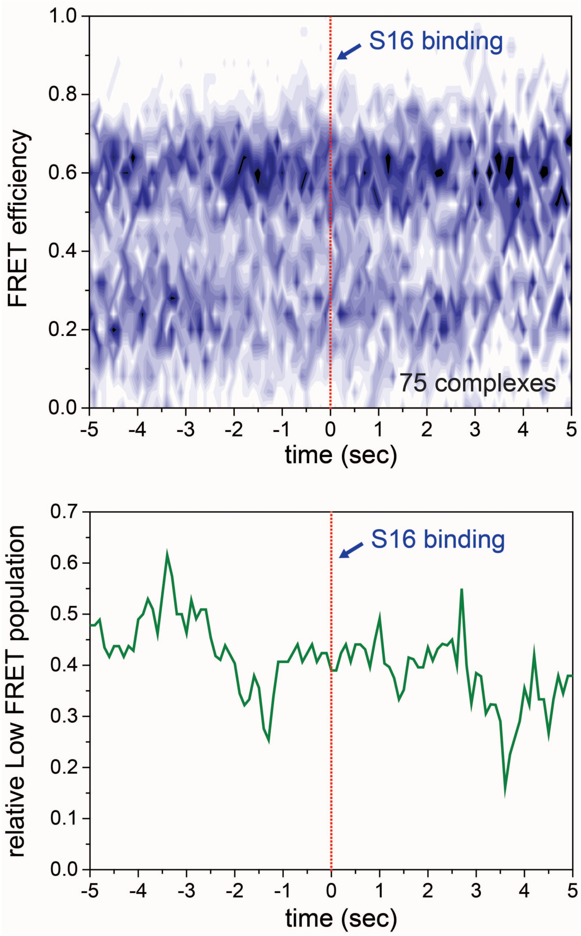

Supplementary Figure 4. Conformation of 5′ domain complexes at S16 binding in 4 mM $MgCl_2$. S16-Cy3 was added to slide chambers containing immobilized complexes of 16S 5′domain-Cy7•S4-Cy3•S20 and imaged as in Figure 4. In 4 mM $MgCl_2$, the low FRET conformation of 16S h3 is more populated, making it easier to detect transitions from the low FRET to high FRET state. (**a**) Time-dependent population map of S4-h3 FRET obtained at 4 mM Mg^{2+}, post-synchronized to the moment of S16 binding (red dashed line). (**b**) Low FRET population ($E < 0.35$) from the map gradually decreases around the moment of S16 binding, similar to the results at 20 mM Mg^{2+}.

SUPPLEMENTARY FIGURE S5

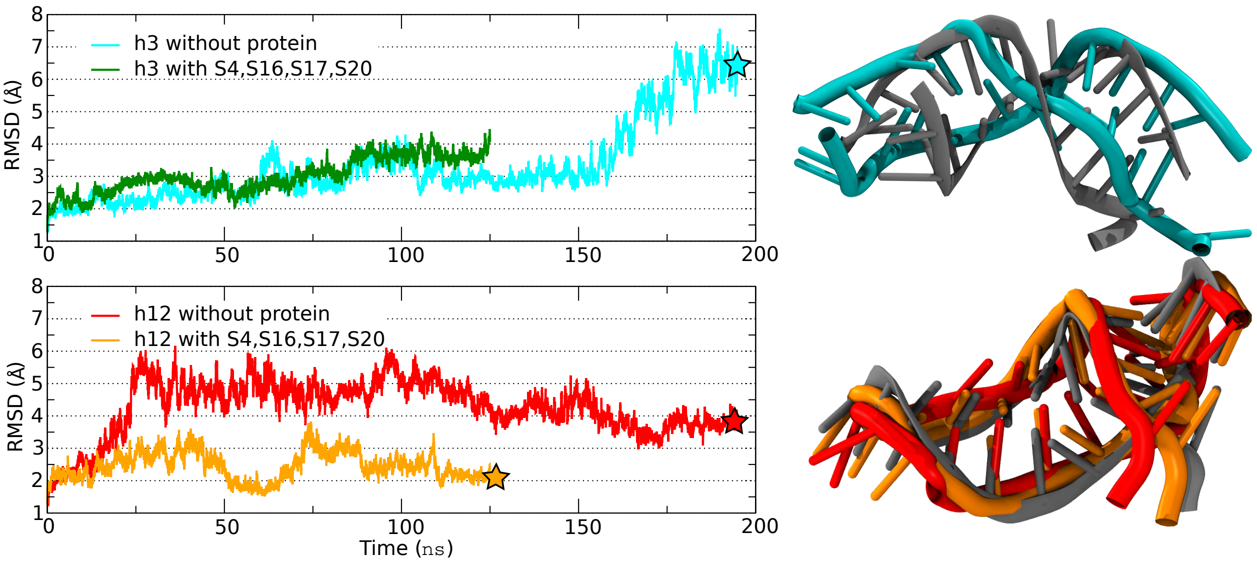

Supplementary Figure 5. Root-mean-squared deviation (RMSD) of h3 and h12 compared to the crystal structure 2I2P. Helices are defined as follows: 27 to 38 and 547 to 556 (h3, cyan); and 289 to 311 (h12, red, orange). Helices are individually aligned to the crystal structure using their heavy atoms. Snapshots from the MD trajectory (stars) and their alignment to the crystal structure helices are also shown.

SUPPLEMENTARY FIGURE 6

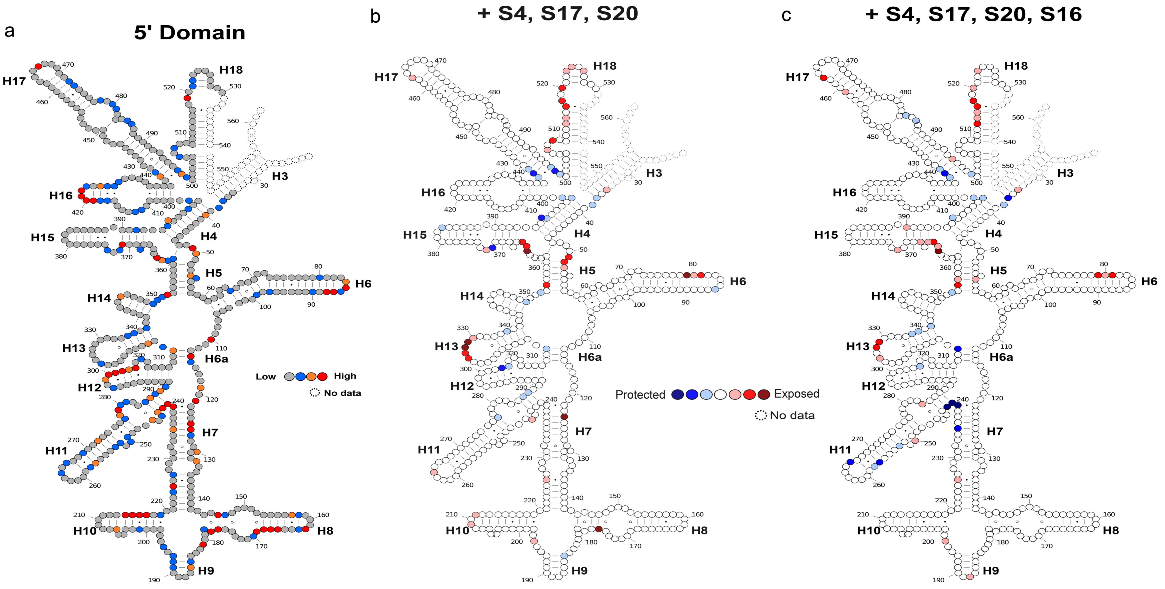

Supplementary Figure 6. SHAPE reactivity of 5′ domain RNA. The secondary structure of the 16S 5′ domain RNA was probed by NMIA (SHAPE) modification as described in Methods. After subtracting the background and discarding outliers (~8σ), the reactivity of each nucleotide was scaled to 100 and clustered. (a) Secondary structure schematic showing relative reactivity of the 5′ domain RNA in 20 mM $MgCl_2$. Nucleotides with SHAPE reactivities greater than 25 are plotted in colors of increasing warmth, non-reactive nucleotides are grey; nucleotides not covered by primer extension reactions are dashed. Except for helix 12, which rearranges during assembly, the SHAPE data are consistent with the 16S secondary structure. (b) Relative SHAPE reactivity (log ratio) in the presence of (b) proteins S4, S17 and S20 or (c) proteins S4, S17, S20 and S16.

SUPPLEMENTARY FIGURE 7

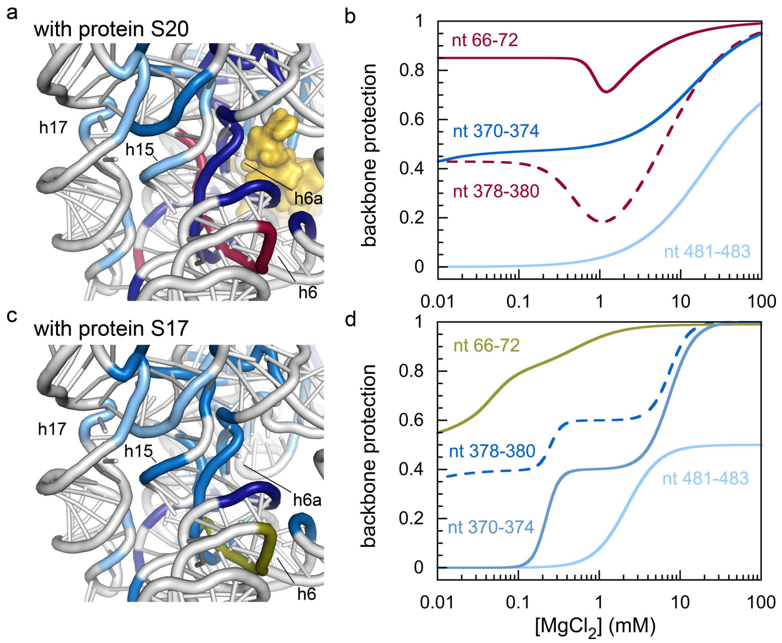

Supplementary Figure 7. Protein S20 drives a conformational switch in 16S h6/6a that permits native interactions with h15 and h17. Hydroxyl radical footprinting of 5' domain RNA bound to S20 (a,b) or S17 (c,d) in 0-20 mM $MgCl_2$, reveals rRNA folding transitions. (a,c) 30S ribosome structure (*2PIP*), showing interactions between h6, h6a, h15 and h17. (b,d) Nucleotides with similar $[Mg^{2+}]_{1/2}$ for folding in Supplementary Ref. [1] were clustered, and saturation of backbone protection for representatives of each cluster are shown as fits to the data. Most RNA residues become more buried and thus more protected at higher $MgCl_2$ concentrations and are shaded blue from the most stable to least stable. In the presence of protein S20, however, nt 66-72 in h6/h6a and nt 378-380 in h15 were buried at low and high $MgCl_2$, but exposed at intermediate $MgCl_2$ concentrations (b, dark pink). These residues do not show switching behavior in the presence of S17 (d, olive).

Supplementary References

1. Ramaswamy, P. & Woodson, S.A. Global Stabilization of rRNA Structure by Ribosomal Proteins S4, S17, and S20. *J Mol Biol*, 666-677 (2009).
